# A new method for rapid comparison of protein binding pockets by capturing spatial distributions

**DOI:** 10.1186/1758-2946-6-S1-P45

**Published:** 2014-03-11

**Authors:** Timo Krotzky, Gerhard Klebe

**Affiliations:** 1 Department of Pharmaceutical Chemistry, Philipps-Universität, Marburg, 35032, Germany

## 

Efficient determination of structural similarities between protein binding pockets is an important challenge in computational chemistry. A degree of similarity in the mutual comparison is often estimated in terms of graphs and by calculating a metric such as the maximum shared common subgraph. Cavbase [1] was developed as a tool for the automatic detection, storage and classification of putative protein binding sites. Cavbase assigns so-called pseudocenters to the cavity-flanking amino acids, which characterize their physicochemical properties with respect to molecular recognition. Subsequently, the pseudocenters are used as graph nodes to accomplish mutual binding site comparisons. This way of modeling protein binding sites, however, tends to be computationally very demanding, which often leads to very lengthy evaluations of the similarity measures.

In this study we propose **Ra**pid **P**ocket **Ma**tching using **D**istances (RAPMAD), a new modeling formalism for Cavbase entries which allows for highly efficient similarity calculations. Here, protein binding sites are represented by sets of distance histograms based on specific spatial reference points [2] in order to characterize the distribution of pseudocenters within the cavity. The histograms can be both generated and compared with linear complexity. Attaining a speed of approximately 20,000 comparisons per second, pocket comparisons across large datasets and even screenings of entire databases become easily feasible.

We demonstrate the discriminative power and the orders of magnitude faster runtime of this novel method by carrying out several classification and retrieval experiments. Among others, datasets of protein cavities hosting specific cofactors are used for classification experiments, where RAPMAD results in a considerably higher rate of correct classifications compared to other alternative approaches while it requires only a fraction of their runtime. Moreover, a set of proteases [3] was investigated, where it turned out that RAPMAD is able to distinguish between different Merops clans such as serine or metallo proteases.
